# Secondary Amenorrhea Revealing a Giant Hamartoma of the Tuber Cinereum

**DOI:** 10.7759/cureus.40532

**Published:** 2023-06-16

**Authors:** Taïeb Ach, Wiem Saafi, Sawsen Nouira, Asma Ben Abdelkrim

**Affiliations:** 1 Endocrinology, Farhat Hached University Hospital, Sousse, TUN

**Keywords:** case report, tuber cinereum, hypothalamus, hamartoma, secondary amenorrhea

## Abstract

Hypothalamic hamartomas are benign tumors composed of ectopic neural and glial tissue. They have a low prevalence and are usually associated with central precocious puberty or epilepsy with gelastic seizures. The presentation beyond childhood is rare, and the symptoms are not the same as in childhood. Here, we report the case of a woman who presented with secondary amenorrhea and headaches revealing a giant hamartoma of the tuber cinereum (TC). The hormonal assessment showed moderate hyperprolactinemia. Synacthen testing was normal. Magnetic resonance imaging revealed a suprasellar hamartoma on the TC measuring 20 mm with sellar extension. The optic chiasma and cavernous sinuses were clear. Hyperprolactinemia was explained by mechanical compression of the pituitary stalk. The patient started cabergoline orally (1 mg per week)* *with an improvement of the prolactin levels and had a natural pregnancy six months later without incident. Surgery was not indicated due to the difficult transsphenoidal access and the absence of major clinical symptoms.

## Introduction

Hamartomas are benign tumors consisting of tissue elements normally found at the same organ site, but growing in a disorganized manner [1]. Hamartomas are mostly asymptomatic incidentalomas that occur in many different parts of the body [2]. The prevalence is one to two cases per 100,000 inhabitants [3]. Cerebral locations of hamartoma are rare, especially those located in the tuber cinereum (TC). The TC is a visible hypothalamic gray matter mass forming the anterior part of the floor of the third ventricle [4]. Tuber cinereum hamartomas are benign gray matter tumors composed of hyperplastic neurons [2]. They are usually asymptomatic small masses that extend into the third ventricle. In some cases, depending on the location, they can cause neurological abnormalities such as cognitive behavioral changes or seizures. Studies have evaluated the occurrence of endocrinological lesions in patients with hypothalamic hamartoma; for example, children may develop clinically early puberty, usually related to the type of parahypothalamic hamartoma in which seizures and developmental delay are not observed [5]. The pathogenesis of these clinical features is still a matter of debate. Immunohistochemical studies show the presence of gonadotropin-releasing hormone (GnRH)-positive neurons in some hypothalamic hamartomas [6]. However, it is assumed that hypothalamic hamartomas do not necessarily induce clinical symptoms as a consequence of their cellular composition but rather of their position relative to hypothalamic structures. Amenorrhea has never been reported as a revealing symptom of TC hamartomas, especially as most of the studies in the literature were based on prepubertal patients. Here, we describe an atypical discovery of a hamartoma of the TC in a young woman who presented with secondary amenorrhea.

## Case presentation

A 23-year-old woman presented to our department with secondary amenorrhea associated with a few headaches. The medical history revealed that the patient had her first period at the age of 19, her menstrual cycle was normal and presented one year after a spaniomenorrhea. Her medical history was significant for recurrent headaches. She had no polyuria or polydipsia.

On examination, there was no hirsutism, no signs of hypothyroidism or hypogonadism with a strictly normal neurological exam. There was no evidence of galactorrhea or abdominal mass. Transvaginal ultrasound showed a normal anteverted uterus. Her blood pressure was 120/76 mmHg. Her weight was 75kg with a body mass index of 30 kg/m². Initial laboratory data revealed a negative human chorionic gonadotropin level.

Biochemical investigations (Table 1) showed normal thyroid function and normal serum levels of growth hormone (GH) and adrenocorticotropic hormone (ACTH). Plasma hormone tests over three weeks including serum concentration of luteinizing hormone (LH), follicle-stimulating hormone (FSH), estradiol, and progesterone, indicated normal cyclical ovarian activity. Hormonal assays identified moderate hyperprolactinemia after dilution of 33 ng/mL (normal range, 3-20 ng/mL). She was not on any medication that explained the hyperprolactinemia.

**Table 1:** Basic hormonal investigations FSH: Follicle stimulating hormone, LH: Luteinizing hormone, TSH: Thyroid stimulating hormone, T4: Thyroxine, GH: Growth hormone, IGF1: Insulin-like growth factor-1, ACTH: Adrenocorticotropic hormone

Hormones	FSH (mUI/mL)	LH (mUI/mL)	Estrogen (pg/mL)	Prolactin (ng/mL)	TSH (mUI/mL)	T4 (pg/mL)	GH (ng/mL)	IGF1 (ng/mL)	ACTH (ng/mL)
Results	10.2	13	115	435	3.5	9	1,5	300	70
Normal range	2.1 - 12	0.8-13	25-200	70-495	0.25-4.5	7-19	1-5	200-400	50-100

Synacthen testing showed a normal adrenal response (cortisol >180 ng/mL). A luteinizing hormone-releasing hormone (LHRH) test was made with 100 μg of LHRH injected intravenously, which showed a normal gonadotrophin response excluding the pituitary cause of the secondary amenorrhea (Table 2). Hyperprolactinemia was the selected diagnosis for secondary amenorrhea after excluding all the other main diagnoses.

**Table 2:** Dynamic hormonal assessment via the LHRH test, where cortisol is measured under Synacthen and gonadotrophin stimulation LHRH: Luteinizing hormone-releasing hormone, FSH: Follicle-stimulating hormone, LH: Luteinizing hormone, T: Time

Test	Hormone measurements	T0 (minutes)	T30 (minutes)	T60 (minutes)	T90 (minutes)	T120 (minutes)
LHRH (100 µg)	FSH (mUI/mL)	6.5	11	12	12.2	12.5
	LH (mUI/mL)	10.7	51	48	50	48
Synacthen (250 µg)	Cortisol (ng/mL)	200	220	260	255	245

**Figure 1:**
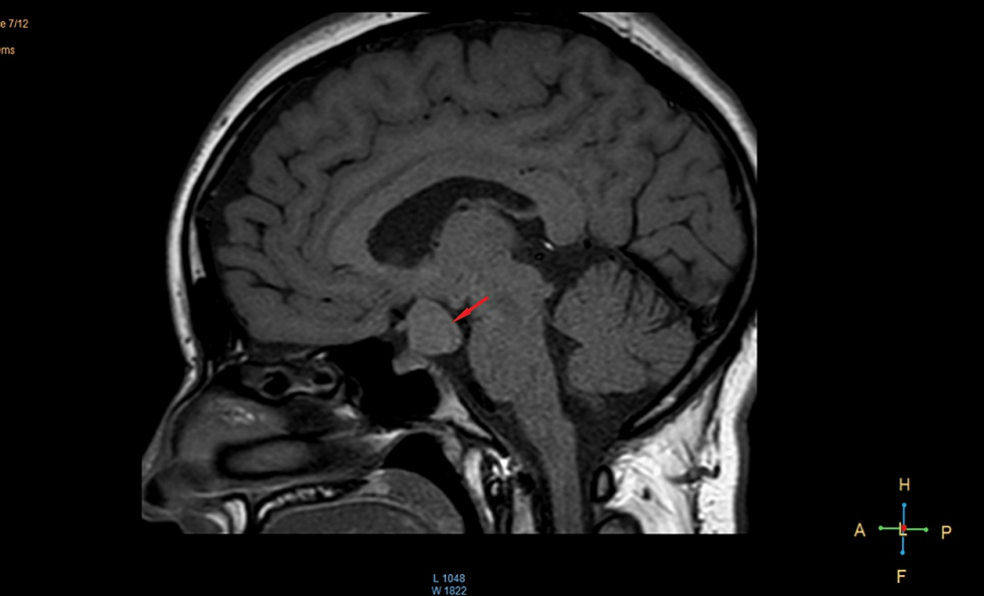
Sagittal view on T1 showing the hamartoma

**Figure 2:**
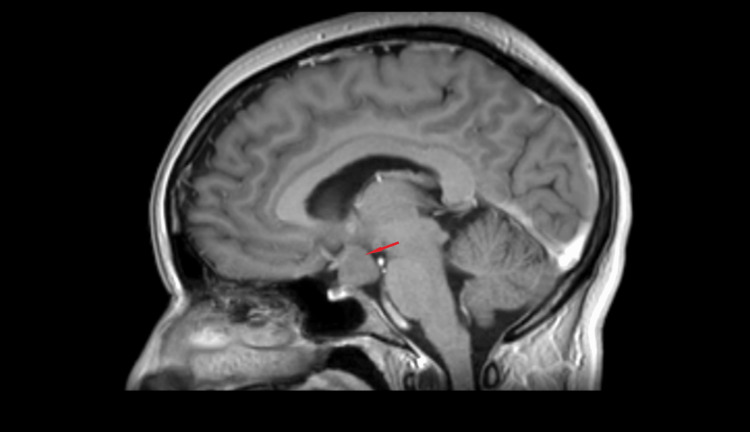
Gadolinium injection in sagittal view on T1

**Figure 3:**
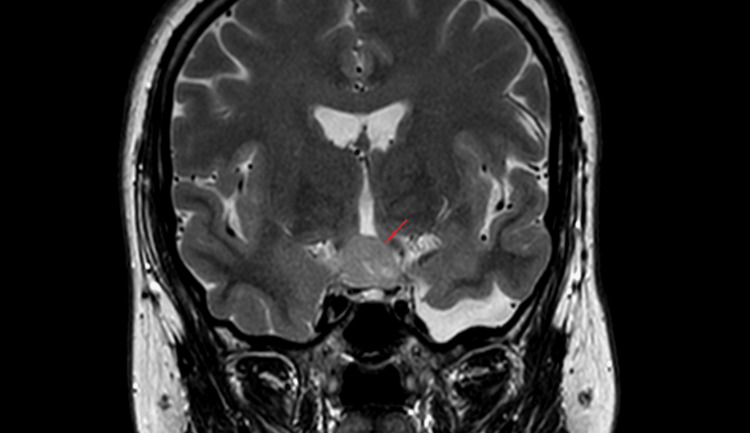
Coronal view reveals the tumoral process developing behind the pituitary gland and in front of the mammillary bodies appended to the TC TC: Tuber cinereum

An MRI revealed a homogenous suprasellar hamartoma of the TC measuring 20 mm, with sellar extension (Figures 1-2). Both the optic chiasm and lateral sinus were clear (Figure 3). The hyperprolactinemia was explained by the mechanical compression of the pituitary stalk.

The patient was started on cabergoline with an improvement of the prolactin levels (20 ng/mL) and had a natural pregnancy six months later without incident. She had spontaneous labor and delivered vaginally a healthy boy weighing 3 Kg. No invasive monitoring was used. Transsphenoidal surgery was not indicated for her hamartoma due to the difficult surgical access and the absence of major clinical symptoms.

## Discussion

Brain hamartomas are benign malformations composed essentially of neurons located in the brain or local cells originating from the site where they develop. They are usually small lesions, between 0.5 cm and 2 cm in diameter, located at the base of the brain on the floor of the third ventricle and in the mammillary bodies [6]. In rare cases, hamartomas can develop in the TC. These lesions may grow slowly in the interpeduncular cistern without displacing adjacent structures. It can take years before signs of compression appear. However, a delay of approximately four years in diagnosing the lesion may explain the size of the hamartoma [7]. Its overall frequency is low, but its importance lies in its association with epilepsy, cognitive-behavioral problems, and/or precocious puberty [2].

In our case, the hamartoma was located in the TC and presented as secondary amenorrhexa. Usually, secondary amenorrhea can be attributed to polycystic ovary syndrome, hypothalamic amenorrhea, hyperprolactinemia, or primary ovarian insufficiency [8]. A patient with mild hyperprolactinemia should undergo a thorough history and physical examination to detect evidence of pituitary compromise, pituitary hypersecretion, or a mass effect. A repeated resting and fasting prolactin level that remains mildly elevated, especially when combined with evidence of pituitary compromise, requires radiologic evaluation of the sella turcica [1].

Pituitary hormones can be disrupted by suprasellar tumors and macroadenomas compressing the pituitary stalk, leading to hyperprolactinemia and hypopituitarism. As a diagnostic tool, MRI is more accurate than CT [9]. It can also be utilized to diagnose hamartoma when no symptoms are present. Pituitary macroadenoma, craniopharyngioma, meningioma, arachnoid cyst, and metastases are among the possible diagnoses for a big sella turcica lesion [10]. Two class III studies examined the presence of endocrine changes in patients with hypothalamic hamartomas and found that potentially 50% of children with hypothalamic hamartomas will develop clinically apparent precocious puberty, commonly associated with the parahypothalamic hamartoma type. Seizures and developmental delays do not seem to occur [5].

The pathophysiology of hamartomas as a cause of secondary amenorrhea, in this case, is due to mechanical pituitary compression. An LHRH test showed a normal gonadotrophin response, and even if this test were normal, the fact that the patient had a pregnancy after normalization of prolactin levels pleads in favor of a pituitary stalk disconnection. Regarding precocious puberty in children, the mechanism involves activation of the secretion of LHRH [7]. Recent studies support the possibility of hypothalamic hamartoma in the production of certain molecules, such as GnRH, transforming growth factor alpha (TGFα), and KISS [11]. In addition, TC hamartomas are not usually associated with other endocrine complications (growth deficiency, diabetes insipidus, etc.), unlike other hypothalamic pathologies [7].

Treatment of TC hamartomas is surgical, mostly via the transsphenoidal approach. All cases pose possible surgical risks (memory loss, polyphagia, diabetes insipidus, and vascular damage) [12]. In our patient, however, transsphenoidal surgery was not recommended due to difficult access and the absence of major clinical symptoms.

## Conclusions

Tuber cinereum hamartoma is a rare, non-neoplastic, heterotopic mass of neural tissue that can be diagnosed incidentally during routine autopsy. In contrast, an early diagnosis is made when clinical symptoms such as precocious puberty and/or gelastic convulsions are present. The pathogenesis of these clinical features is still a matter of debate. In our case, the main symptom was secondary amenorrhea. The position of a hypothalamic hamartoma and involvement of the third ventricle are likely to be more predictive of clinical characteristics than size and shape. Surgery is not always needed, as in the case of our patient, especially with difficult transsphenoidal access and the absence of major clinical symptoms.
